# PRESERFLO™ Microshunt: 1-Year Results of a 25-Gauge vs. 27-Gauge Needle Tract

**DOI:** 10.3390/jcm13071979

**Published:** 2024-03-29

**Authors:** Stefan Steiner, Hemma Resch, Barbara Kiss, Clemens Vass

**Affiliations:** 1 Department of Ophthalmology, Medical University of Vienna, 1090 Vienna, Austria; 2 Department of Ophthalmology, Sanatorium Hera, 1090 Vienna, Austria

**Keywords:** PreserFlo, microshunt, MIGS, glaucoma surgery, filtering surgery, glaucoma

## Abstract

**Background:** The purpose of this study was to evaluate the effectiveness and safety of the PreserFlo™ microshunt (PMS) using a 25-Gauge vs. 27-Gauge needle tract. **Methods**: This is a prospective postoperative examination of 60 glaucoma eyes that received a PMS. The main outcome measures were intraocular pressure (IOP), glaucoma drug score (GDS), Kaplan–Meier success rates, complications, and secondary intervention rates. Two subgroups were formed for data comparison: 27-Gauge (27G), and 25-Gauge (25G). Success was defined as IOP < 18 mmHg together with ≥20% IOP reduction with medication allowed (qualified success = QS18) or not (full success = FS18). **Results**: IOP and GDS were reduced from baseline to the 1-year study visit as follows: All eyes from 23.4 ± 8.6 mmHg (3.1 ± 0.9) to 15.1 ± 5.9 mmHg (0.8 ± 1.1); 25G from 24.2 ± 7.3 mmHg (3.0 ± 0.8) to 12.7 ± 2.7 mmHg (0.5 ± 0.8); and 27G from 23.1 ± 9.2 mmHg (3.1 ± 1.0) to 16.2 ± 6.7 mmHg (0.9 ± 1.2). IOP at one year was lower in the 25G group compared to the 27G group (*p* = 0.035). Bleb needling was required in eight (13.3%) eyes and open bleb revisions in three (5.0%). Transient hypotony occurred in 21% and choroidal effusion in 8% of all eyes. Choroidal effusions were more frequent in the 25G group (21%) compared to the 27G group (2%, *p* = 0.031). One-year success rates were significantly higher in the 25G group compared to the 27G group for both QS18 (25G: 67.9% vs. 27G: 35.7%, *p* = 0.002) and FS18 (25G: 63.6% vs. 27G: 29.2%, *p* = 0.007). **Conclusions**: The PreserFlo microshunt is an effective and safe glaucoma surgery with a low rate of bleb revisions or needlings. We show that the 25G needle tract might be more efficient for IOP control at the cost of increased IOP-related complications compared to 27G.

## 1. Introduction

The main treatment goal for glaucoma is to stop disease progression. This can be achieved by lowering the intraocular pressure (IOP), which is the main modifiable risk factor for glaucoma [1]. IOP can be lowered by using IOP-lowering eye drops, laser interventions, or glaucoma surgery. Due to poor IOP-lowering effects, intolerances, allergies, or patient compliance [2], eye drops are not always sufficient for glaucoma treatment and a surgical approach may be needed. Trabeculectomy is the most commonly performed glaucoma surgery achieving good IOP reduction by subconjunctival filtration. This procedure has undergone multiple improvements since its introduction, leading to decreased complication rates [3]. Additionally, several other types of glaucoma surgery including minimally invasive glaucoma surgery (MIGS) have been introduced in the past decades, mostly to reduce the perioperative risk and provide rapid rehabilitation of the patient. These procedures usually use alternative, more natural outflow pathways to Schlemm’s canal or the supraciliary space. However, in general, there has been some trade-off between the safety and efficacy of these procedures [4,5]. More recently, the implantation of the PreserFlo™ microshunt (PMS) is an emerging surgery which aims at subconjunctival filtration in a controlled manner. PMS is expected to offer an improved safety profile and more standardized surgery and may achieve IOP values close to those after traditional trabeculectomy [6,7,8,9,10,11,12]. Similar to the XEN-45 implant [13], the PMS tube shunt drains the aqueous humor to a conjunctival filtering bleb. However, unlike the XEN-45, the PMS is made of poly(styrene-block-iso-butylene-block-styrene), also known as SIBS [14], instead of gelatine, an established material used for cardiac stents with virtually no foreign body reaction, to minimize tissue reaction and prevent scarring of the filtering bleb [6]. In the present study, we report the one-year results of the PMS with the goal to investigate differences in a 25-Gauge vs. 27-Gauge needle tract approach.

## 2. Materials and Methods

This is an observational cohort study with a prospective postoperative examination of glaucoma patients who received a PMS (ClinicalTrials.gov Identifier: NCT04541524). The target population consisted of patients who underwent PMS implantation, either as a standalone procedure or in combination with cataract extraction, between January 2019 and November 2019 at the Department of Ophthalmology, Medical University of Vienna, Austria. Patients were invited to participate in a postoperative examination 12 (±4) months after surgery. No specific inclusion criteria were defined; therefore, all patients (as seen in the target population) were asked to participate in the examination one year after PMS implantation. Patients meeting any of the following exclusion criteria were not included: unwilling or unable to give informed consent; pregnancy or lactation. Apart from the prospective 1-year visit, baseline data and data prior to the 1-year visit were collected retrospectively. This study was approved by the local ethics committee and followed the tenets of the Declaration of Helsinki. Informed consent was obtained from all subjects involved in this study. 

As part of our routine procedure, all eyes were switched to preservative-free medication and all eyes received hydrocortisone eye drops topically for the last two weeks prior to surgery. The PMS was implanted via an ab externo approach similar to the description of Pinchuk et al. [6]. Preoperatively, the eye was disinfected, and topical anesthesia was applied using 4% lidocaine eye drops. Approximately 8 min prior to the creation of the conjunctival flap, 20 µg of Mitomycin C (MMC) in a 0.1 mL solution (0.2 mg/mL) was injected under the conjunctiva at the planned surgical location, which was mostly in the supero-temporal quadrant or, in rare cases, in the supero-nasal quadrant or inferiorly. This was followed by digital massage to ensure a wide spread of the bolus. At the surgeons’ discretion, we had some variations on the surgical technique. The conjunctival flap was created either fornix-based (limbal conjunctival opening) or limbus-based (conjunctival opening 3.5 mm from the limbus). The Tenons layer was carefully dissected from the sclera towards the posterior segment using a blunt scissor forming a deep and wide Tenon pocket. A one-millimeter wide and long scleral pocket was made at a 3.0 or 3.5 mm distance from the surgical limbus in which the PMS was introduced via a 25- or 27-Gauge needle tract to the anterior chamber. The position of the needle and/or the PMS after implantation was checked with a 4-mirror gonio lens. In case of inappropriate position, a new scleral pocket and needle tract was formed adjacent to the prior one. As soon as aqueous humor flow through the PMS was confirmed, the conjunctival flap and the Tenons layer was sewed either together or separately at the discretion of the surgeon. In cases of combined operations, phacoemulsification was performed before the implantation of the PMS. The method varied slightly among surgeons. In one approach, a clear corneal incision (CCI) was positioned in the upper right quadrant before opening the conjunctiva, followed by cataract surgery, with the PMS subsequently implanted in the upper left quadrant. In the other approach, the conjunctiva was opened first, followed by the subconjunctival limbal cataract wound in the upper right quadrant and cataract operation. The PMS was then implanted temporally in the 11 o’clock position adjacent to the cataract wound.

If indicated, open filtering bleb revision was performed in the operating room with the same technique used for open XEN bleb revision described in our study [15]. Needlings were performed at the slit lamp or in the operating room using a bent needling knife (Kai Corporation, Tokyo, Japan), followed by subconjunctival injection of 0.1 mL of a 0.2 mg/mL MMC solution.

The main outcome measures were pre- and postoperative intraocular pressure (IOP), glaucoma drug score (GDS), Kaplan–Meier success rates, complications, and secondary intervention rates. The GDS was defined by the number of different active ingredients of the topical eye drops with an extra point for the administration of acetazolamide. Initially, we evaluated two surgical variations for their effect on the outcomes, the needle tract size (25G vs. 27G) and the conjunctival opening (fornix based vs. limbus based). Subgroups were determined according to results of a multivariate Cox proportional hazards model. Since the fornix vs. limbus-based filtering bleb approach showed no significant effect on the success rates of the PMS implantation, we decided to present only the 25-Gauge vs. 27-Gauge approach in the subgroup analysis. Therefore, two subgroups were formed for data comparison: 25-Gauge (25G) and 27-Gauge (27G). 

Patient data were retrieved retrospectively from patient files and were categorized in the following time periods: D1 (day 1–2), W1 (day 3–8), W2 (day 9–15), M1 (day 16–60), M3 (day 61–122), M6 (day 123–272), and Y1 (day 273–547). Up to three of the most recent preoperative data records which determined the surgical indication were considered for data analysis.

Qualified success (QS, glaucoma medication allowed) and full success (FS, no glaucoma medication allowed) was defined as an IOP reduction of 20% or more together with an IOP below 21 mmHg (QS21, FS21) or below 18 mmHg (QS18, FS18). No lower IOP limit was set for failure.

A Kaplan–Meier survival analysis was carried out to compare the failure rates of the 25G and 27G groups with the success criteria mentioned above. Failure was determined as not meeting the mentioned criteria on two consecutive visits after 60 days of follow-up or at the last visit, or requiring needling, filtering bleb revision, or any other secondary glaucoma surgery.

Any occurrence of an IOP below 6 mmHg lasting less than 2 months and not followed by other complications was recorded as transient hypotony. In case of additional complications like choroidal detachment or shallowing of the anterior chamber, we recorded the event as clinically relevant hypotony.

Statistical analyses were performed using SPSS^®^ (Version 21, IBM Corp., Armonk, NY, USA). Differences in baseline characteristics were compared between the subgroups using unpaired *t*-tests for numeric variables and Chi-squared tests for frequencies. One-Way ANOVA was used to compare pre- and postoperative IOP and GDS between subgroups. Success rates in our Kaplan–Meier analyses were compared using the Logrank test pairwise over strata. A multivariate Cox proportional hazards model was calculated to stratify baseline risk factors for failure. All tests were two-sided with *p* values < 0.05 considered as statistically significant.

## 3. Results

We successfully recruited and included a total of 60 eyes (52 patients) in the analyses, which was 83.3% (*n* = 60/72) of the target population. None of the patients had to be excluded. Some patients were unwilling to participate, primarily due to the ongoing COVID-19 pandemic at that time. Among these 60 eyes, PMS implantation combined with phacoemulsification was performed in nine eyes, with three in the 25G subgroup and six in the 27G subgroup (*p* = 0.907). The baseline characteristics of the participants are shown in Table 1. There were no statistically significant differences between the two subgroups for the baseline characteristics.

**Figure 1:**
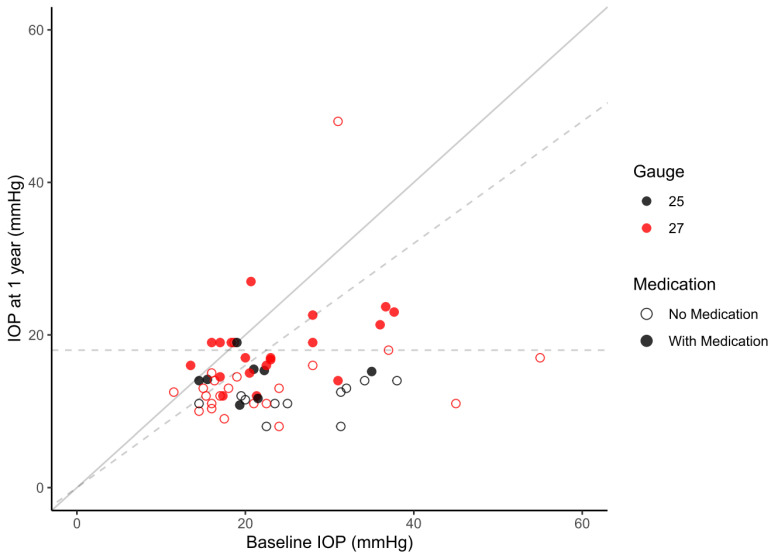
Scatter plot of preoperative IOP vs. IOP at the one-year study visit. Dots are color-coded according to the subgroups. Patients not on medication after one year are indicated by empty circles, with medication by solid circles. The solid diagonal line indicates matching baseline IOP and IOP at 1 year, meaning that all points below the line indicate a reduction in IOP, while those above represent higher IOP values at 1 year compared to baseline. The dashed horizontal line marks a postoperative IOP of 18 mmHg and the dashed diagonal line marks an IOP reduction of 20%.

Details of the course of IOP and GDS are shown in Table 2. The mean IOP (GDS) at 1-year postoperative was 15.1 ± 5.9 mmHg (0.8 ± 1.1) for the whole cohort, 12.7 ± 2.7 mmHg (0.5 ± 0.8) in the 25G subgroup, and 16.2 ± 6.7 mmHg (0.9 ± 1.2) in the 27G subgroup. All groups showed a statistically significant reduction in IOP and GDS after PMS implantation at each given timepoint compared to the baseline values. A scatterplot of preoperative IOP vs. IOP at the one-year study visit are shown Figure 1. IOP reduction was achieved in 88.3%, 83.3% had an IOP of less than 18 mmHg, and IOP was reduced by 20% or more in 73.3% of the study eyes after 1 year of follow-up. At the same time, 93.3% of the included eyes had a lower GDS, 5.0% were the same, and 1.7% had a higher GDS compared to preoperatively. The percentage of eyes that were free from medication at one year was 66.7%, and the mean IOP of this subgroup was 13.3 mmHg, a mean reduction of 55.0% from the baseline.

The intergroup comparison between the 25G and 27G subgroups revealed no significant differences in baseline IOP and GDS. IOP at the 1-year follow-up was significantly lower in the 25G group compared to the 27G group. No differences in IOP were observed at other timepoints between groups. GDS was only significantly higher in the 27G group compared to 25G group at the 6-month mark.

Needling was required in eight eyes (13.3%), while three eyes underwent open bleb revision (5%). Among the eyes that underwent needling, one required an additional open bleb revision. No statistically significant difference in the frequency of needlings or open bleb revisions was observed between our subgroups. Two of nineteen eyes in the 25G group (one needling, one bleb revision) and eight of forty-one eyes (seven needlings, two bleb revisions) in the 27G group needed an IOP-related secondary intervention. Overall, 87.5% of the needled and 100% of the bleb-revised eyes reached a postoperative IOP of less than 21 mmHg and 20% IOP reduction. Two of three eyes after bleb revision and three of seven eyes after needling were free from glaucoma medication one year after PMS implantation.

**Figure 2:**
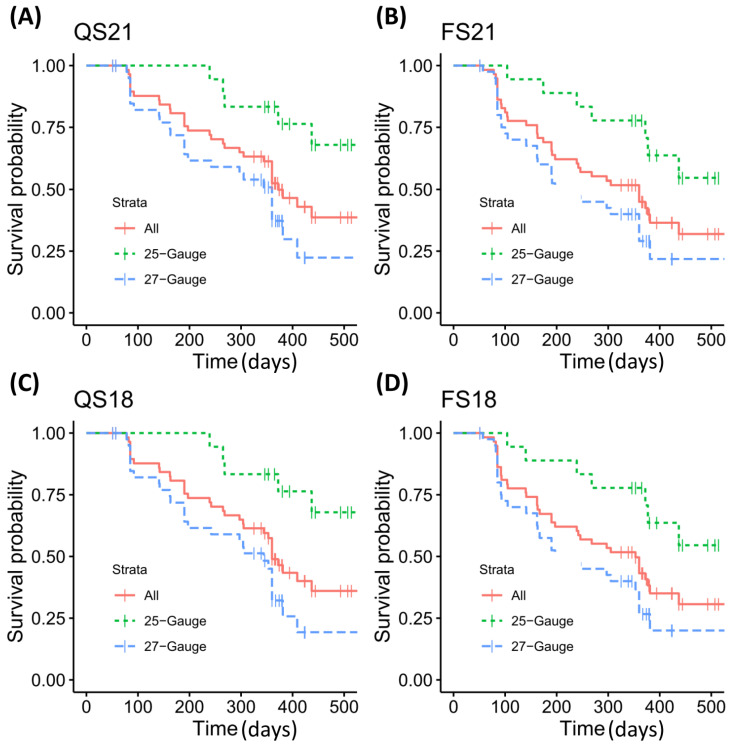
Kaplan–Meier plots comparing success rates of the full cohort and the 25–Gauge and 27–Gauge subgroups; (**A**) QS21 = qualified success with postoperative IOP < 21 mmHg together with ≥20% IOP reduction with medication allowed; (**B**) FS21 = full success with postoperative IOP < 21 mmHg together with ≥20% IOP reduction with no medication allowed; (**C**) QS18 = qualified success with postoperative IOP < 18 mmHg together with ≥20% IOP reduction with medication allowed; (**D**) FS18 = full success with postoperative IOP < 18 mmHg together with ≥20% IOP reduction with no medication allowed.

Kaplan–Meier survival curves of the whole cohort and subgroups using the QS21, QS18, FS21, and FS18 criteria are shown in Figure 2. Additionally, corresponding mean survival times are shown in Table 3. In the whole cohort, the Kaplan–Meier success rates (standard error) at 1 year were 51.6% (6.8%), 48.1% (6.8%), 44.2% (6.7%), and 42.4% (6.7%) for the QS21, QS18, FS21, and FS18 criteria, respectively. The subgroup analysis revealed a statistically significant longer survival in the 25G group compared to the 27G group.

Comparing eyes that received a standalone PMS implantation with PMS implantation plus phacoemulsification and intraocular lens implantation showed no differences in Kaplan–Meier survival time in any of the success criteria. The mean survival times are shown in Table 3.

Hazard ratios of the Cox proportional hazards models for the success criteria QS21 and FS18 are shown in Table 4. To keep the overview of the table, we did not report on the QS18 and FS21 success criteria, since they provided comparable results. The approach with a 25-Gauge needle tract was strongly associated with a lower risk of failure in the QS21 and FS18 success criteria. Patients who underwent other glaucoma surgery prior to PMS implantation tended to have an increased risk of failure, although this result was only significant for the FS18 criterion and borderline significant for the QS21 criterion. A possibly protective covariate was the category of “Diagnosis: Other” (containing angle closure glaucoma and secondary glaucoma other than PEX or pigmentary glaucoma), which was again significant for the FS18 criterion and only borderline for the QS21 criterion. All other suspected factors, including combined PMS implantation with cataract extraction, were not statistically significant.

Complications are listed in Table 5. Transient numerical hypotony was the most frequently observed complication after PMS implantation and resolved mostly without intervention (92%) after a median time of 12 days and a maximum of 39 days. The median duration of choroidal effusions was 39 days, of which three of five cases with choroidal effusion lasted longer than 30 days. One choroidal effusion, which was clinically classified as choroidal bleeding, lasted a total of 428 days and was last visible only as a small-to-medium-sized buckle. Only in two of five eyes that suffered from choroidal effusion was hypotony detected. Clinically relevant hypotony, choroidal effusions, and the compound outcome of at least one of the recorded complications occurred in a statistically significant higher rate in patients with a 25G needle tract compared to 27G (see Table 5). No statistical differences were found between the given subgroups for other complications. No cases of corneal decompensation were observed during the follow-up period. Nine eyes (15%) experienced a loss in visual acuity of two lines or more. No adverse events like endophthalmitis, retinal detachment, malignant glaucoma, or loss of light perception were recorded.

## 4. Discussion

The key finding of this study is that the PreserFlo™ microshunt yields a high full success rate of 63.6% at 1-year follow-up, with a IOP reduction of more than 20% and below 18 mmHg, but only when the implant was inserted via a 25G needle tract. Surprisingly, the group with the 27G needle tract performed markedly worse and presented a less than 30% success rate for the same success criteria. The dependence of success on the needle size in our data was confirmed by the multivariate Cox proportional hazards model, where the use of a 27G needle tract compared with the 25G one was a significant predictor of failure in our data.

The poorer performance of the 27G needle tract was surprising for us, since the diameter of this needle (412.8 µm) matches the outer diameter of the implant better (350 µm), while the 25G needle (514.4 µm), in theory, leaves some space around the implant. During the initial phase in January 2019, we started by using a 25G needle, but we did encounter a few complications from early ocular hypotony. We suspected flow around the tube as a possible cause, which prompted us to use the tighter-fitting 27G needle, as it had been used by Batlle [9]. We then experienced good initial results with only rare cases of transient numerical hypotony. Towards the end of 2019, we started noticing patients with rising IOP, which we attributed initially to the limbus-based approach, which one of us (CV) had adopted at the same time when changing to the 27G needle. Only the prospective 1-year follow-up examination, together with retrospective gathering and analysis of data from patient files, revealed the needle diameter as the likely responsible factor in our data, while the type of the conjunctival opening proved non-significant. 

What might be the reason for the worse performance of the 27G needle tract? We speculate that, in some subjects, ongoing scarring processes of the sclera might compress the PMS, and that a narrower needle tract might increase the likelihood of this process. This compression would then increase the resistance within the PMS, which adds to the distal resistance within the bleb and ultimately increases the IOP.

Our results are comparable with the published literature, where the mean IOP and medication were reduced from an average of 20.1–25.1 mmHg at 2.0–3.5 glaucoma medication at baseline to 10.7–14.7 mmHg at 0.2–0.9 at 1-year [7,8,9,10,16,17,18,19,20,21,22], even though, in our data, the mean IOP was on the upper end of the spectrum, with 15.1 mmHg at 0.8 medications one year postoperatively for the whole cohort. Contrary to that, our results of the 25G eyes compared perfectly well with already published data, with a mean IOP of 12.7 mmHg at 0.5 medications. Similarly, the success rates at an IOP below 18 mmHg of the mentioned studies were between 58% and 77% full success at 1 year, compared to 44% in our complete series [7,8,20]. However, the subgroup with the 25G needle tract performed well, with full success below 18 mmHg in 64% of cases. It is important to note that our success criteria were somewhat stricter because any revision procedure, including needling (13%), was considered a failure, whereas in the cited literature, needling procedures were not regarded as a failure, and have been performed in 5% to 20% of cases. 

To the best of our knowledge, a possible effect of the needle diameter used on the success rates or the IOP values has not yet been published. Looking at the present literature on surgical methods, the results are heterogeneous. In their small prospective study, Batlle et al. [9,10] used 27G needles for 20 patients and 25G needles for 3 patients, and reported one-year postoperative IOP of 10.7 mmHg. This study was performed in the Dominican Republic, and the cohort studied there was probably not comparable to our situation for ethnic reasons, but maybe also because of differences in age and duration of previous treatment. Furthermore, Batlle included only POAG patients, and 39% of the procedures were combined with phacoemulsification. In some cohorts, a 1 mm microknife [8,20] or a special double-step microknife was used to create both the scleral pocket and the tunnel to the anterior chamber angle [17]. All other case series used 25G needle tracts to implant the PMS [7,16,18,21,22,23,24] or did not report on the needle dimension [12]. Contrary to our findings, the current literature does not support an association between needle dimension and success rates or IOP at the one-year postoperative mark. When calculating weighted means considering the sample sizes of publications reporting on 1-year postoperative results [7,8,9,17,18,20,24], the mean IOP is 14.2 mmHg for a 27G needle, 13.8 for a 25G needle, and 13.9 for a microknife. However, the evidence regarding the 27G needle tract is extremely heterogeneous. Except for our own data, which recorded a one-year postoperative IOP of 16.1 mmHg in 41 eyes treated with a 27G needle tract, the most comparable results come from a small initial study by Batlle [9]. This study reported the most favorable outcomes among all investigations, with a mean IOP of 10.7 mmHg in 23 eyes (20 with a 27G needle tract).

Only a small number of eyes required further surgical interventions in our cohort (13.3% needlings, 5.0% open bleb revisions). This low revision/needling rate was also confirmed in other studies (4.0–20% needlings) [7,8,9,10,16,17,20,21,22], especially in comparison to the most similar subconjunctival MIGS, XEN-45, with needling and revision rates of 22.1–49.6% [15,25,26,27,28,29,30,31]. This tendency towards less postoperative interventions for the PMS compared to the XEN-45 was also observed by Scheres et al. [7], although they did not find significant differences in success, intraocular pressure, and medication. When we compare the qualified success rates of IOP below 21 mmHg of our current microshunt study (all subgroups together) with those of our XEN-45 study [15], calculated with the same method considering needlings and bleb revisions as failures, we find a slight difference in favor of the PreserFlo™ microshunt, with 51.6% in PMS vs. 37.7% in XEN-45 after one year of follow-up. 

In our cohort, PMS implantation was followed by only a small number of complications, with significantly fewer IOP-related complications in the 27G subgroup. The rate of hypotony (21%) and choroidal effusions (8%) was consistent with other studies where the rate of postoperative self-limiting hypotony was between 13 and 69%, and that of choroidal detachments was 2–15% [7,8,9,10,11,16,20,21,22]. These findings suggest a potential trade-off between achieving stronger IOP-lowering effects and the increased risk of postoperative IOP-related complications.

There are several limitations to our study. Firstly, our study cohort is notably heterogeneous, comprising patients with varying characteristics and surgical approaches. Specifically, we included patients undergoing PMS implantation with 27G or 25G tracts, with and without concurrent phacoemulsification, and utilizing either fornix- or limbal-based conjunctival flaps. Additionally, the procedures were performed by three different surgeons, each at different stages of their learning curves. Furthermore, the patients had diverse histories, including previous glaucoma operations and varying etiologies of glaucoma. Given that our data, except for the one-year results, are of a retrospective nature, our study’s results do not prove a causal relationship between needle diameters and the efficacy and safety profile of the PMS. However, the results can be cautiously interpreted as an association, pending independent confirmation.

## 5. Conclusions

The PreserFlo™ microshunt is an effective and safe glaucoma surgery with a low rate of reinterventions, like bleb revision or needling. To the best of our knowledge, we are the first group comparing a 25G vs. 27G needle tract approach. In the present study, we show that the 25G needle tract might be the more efficient method for IOP control, albeit at the price of increased IOP-related complications.

## Figures and Tables

**Table 1:** Baseline characteristics.

	All	25-Gauge	27-Gauge	*p*-Value
	*n* = 60	*n* = 19	*n* = 41	
Age (years). median (IQR)	72 (58–79)	74 (57–81)	71 (58–79)	0.251
Sex. *n* (%)				0.511
Female	29 (48)	8 (42)	21 (51)	
Eye. *n* (%)				0.896
Left	34 (57)	11 (58)	23 (56)	
Glaucoma type. *n* (%)				0.635
Primary open-angle glaucoma	29 (48)	10 (53)	19 (46)	
Pseudoexfoliative glaucoma	15 (25)	5 (26)	10 (24)	
Pigment dispersion glaucoma	4 (7)	0 (0)	4 (10)	
Primary angle-closure glaucoma	1 (2)	0 (0)	1 (2)	
Other *	11 (18)	4 (21)	7 (17)	
History of glaucoma surgery. *n* (%)	13 (22)	2 (11)	11 (27)	0.154
Trabeculectomy	8 (13)	1 (5)	7 (17)	
Xen	5 (8)	1 (5)	4 (10)	
Lens status. *n* (%)				0.967
Pseudophakic	16 (27)	5 (26)	11 (27)	

* Other glaucoma types included: secondary glaucoma after eye surgery/infection (*n* = 2), neovascular glaucoma (*n* = 1), aphakic glaucoma (*n* = 3), congenital glaucoma (*n* = 2), cortison response (*n* = 1) and ocular hypertension (*n* = 2). No statistical differences were observed between the subgroups. The t-test for independent samples was used to compare continuous variables and chi-square test was used for categorical variables. IQR = interquartile range.

**Table 2:** Intraocular pressure (IOP) and glaucoma drug score (GDS) over time.

		All *n* = 60	25-Gauge *n* = 19	27-Gauge *n* = 41	*p*-Value
Pre OP	IOP (mmHg)	23.4 ± 8.6	24.2 ± 7.3	23.1 ± 9.2	0.639
	GDS	3.1 ± 0.9	3.0 ± 0.8	3.1 ± 1.0	0.701
	*n*	60	19	41	
Day 1–2	IOP (mmHg)	10.6 ± 7.0	10.1 ± 4.3	10.9 ± 8.2	0.717
	GDS	0.1 ± 0.5	0.0 ± 0.0	0.2 ± 0.6	0.247
	*n*	49	18	31	
Week 1	IOP (mmHg)	11.6 ± 5.7	10.1 ± 3.5	12.2 ± 6.3	0.238
	GDS	0.1 ± 0.6	0.0 ± 0.0	0.2 ± 0.7	0.283
	*n*	49	15	34	
Week 2	IOP (mmHg)	11.3 ± 6.7	10.2 ± 4.8	11.7 ± 7.4	0.499
	GDS	0.2 ± 0.7	0.0 ± 0.0	0.3 ± 0.8	0.264
	*n*	42	13	29	
Month 1	IOP (mmHg)	12.1 ± 4.4	10.6 ± 3.3	12.8 ± 4.7	0.072
	GDS	0.1 ± 0.4	0.1 ± 0.2	0.2 ± 0.5	0.315
	*n*	58	18	30	
Month 3	IOP (mmHg)	12.4 ± 3.2	11.2 ± 3.0	13.0 ± 3.2	0.061
	GDS	0.2 ± 0.6	0.0 ± 0.1	0.3 ± 0.7	0.192
	*n*	52	16	36	
Month 6	IOP (mmHg)	14.3 ± 4.0	13.5 ± 4.2	14.8 ± 3.8	0.267
	GDS	0.6 ± 1.0	0.3 ± 0.7	0.8 ± 1.1 *	0.046
	*n*	48	17	31	
Year 1	IOP (mmHg)	15.1 ± 5.9	12.7 ± 2.7	16.2 ± 6.7 *	0.035
	GDS	0.8 ± 1.1	0.5 ± 0.8	0.9 ± 1.2	0.137
	*n*	60	19	41	

* Significant difference between subgroups (*p* < 0.05). The one-way ANOVA was used. IOP = Intraocular pressure, GDS = glaucoma drug score, 25G or 27G = Gauge number for needle diameter.

**Table 3:** Mean survival time (95% Confidence interval) in days.

	Overall	25-Gauge	27-Gauge	*p*-Value	Stand-Alone Surgery	Combined Surgery	*p*-Value
	Mean Survival	Mean Survival	Mean Survival		Mean Survival	Mean Survival	
	*n* = 60	*n* = 19	*n* = 41		*n* = 51	*n* = 9	
QS21	359 (313–404)	448 (385–511)	308 (254–361) *	0.005	344 (295–393)	435 (339–532)	0.192
QS18	352 (308–397)	448 (385–511)	300 (250–352) *	0.002	340 (292–389)	421 (327–515)	0.296
FS21	315 (268–361)	398 (330–467)	270 (215–325) *	0.010	299 (251–347)	377 (259–496)	0.274
FS18	311 (265–357)	396 (327–466)	266 (212–319) *	0.007	298 (249–346)	361 (248–475)	0.455

* Significant difference between subgroups (*p* < 0.05) using log-rank test; QS21/FS21 and QS18/FS18 = postoperative IOP < 21 mmHg and <18 mmHg together with ≥20% IOP reduction; QS = Qualified success with medication allowed; FS = Full success with no medication allowed.

**Table 4:** Hazard ratios for not meeting the given success criteria *.

Covariates		QS21		FS18	
	*n*	Hazard Ratio (95% CI)	*p*-Value	Hazard Ratio (95% CI)	*p*-Value
Age	60	1.02 (0.98–1.07)	0.308	1.02 (0.98–1.06)	0.274
Sex: Male	31	1.00 (0.43–2.30)	0.994	0.82 (0.39–1.73)	0.596
Baseline IOP	60	1.03 (0.96–1.11)	0.452	1.04 (0.97–1.12)	0.244
Baseline number of glaucoma medication	60	0.85 (0.50–1.45)	0.560	0.95 (0.58–1.55)	0.828
Diagnosis: Primary open angle	28	Reference		Reference	
Diagnosis: PEX	15	0.72 (0.23–2.28)	0.579	0.60 (0.20–1.79)	0.356
Diagnosis: Pigmentary glaucoma	4	3.48 (0.63–19.17)	0.152	2.92 (0.60–14.12)	0.183
Diagnosis: Other	12	0.37 (0.13–1.03)	0.058	0.34 (0.13–0.92)	0.033
Glaucoma surgery prior to PMS	13	2.42 (0.97–6.08)	0.059	2.65 (1.12–6.23)	0.026
Combined PMS with cataract extraction	9	0.47 (0.13–1.70)	0.248	0.81 (0.28–2.32)	0.692
Conjunctival opening: Limbus based	23	1.23 (0.46–3.33)	0.678	1.11 (0.45–2.76)	0.815
Needle diameter: 25G	19	0.47 (0.24–0.90)	0.022	0.48 (0.27–0.86)	0.013
Number of created tunnels	60	1.28 (0.76–2.14)	0.360	1.12 (0.67–1.87)	0.658

* Hazard ratios calculated using Cox proportional hazards model; IOP = Intraocular pressure, PEX = pseudoexfoliation, PMS = PreserFlo microshunt; QS = qualified success with medication allowed; FS = full success with no medication allowed.

**Table 5:** Complications, *n* (%).

	All	25-Gauge	27-Gauge	*p*-Value
	*n* = 60	*n* = 19	*n* = 41	
Transient ocular hypotension	13 (21)	7 (37)	6 (15)	0.089
Clinically relevant ocular hypotension	4 (7)	4 (21)	0 (0) *	0.008
Shallowing of the anterior chamber	1 (2)	1 (5)	0 (0)	0.317
Choroidal effusion	5 (8)	4 (21)	1 (2) *	0.031
Wound dehiscence	1 (2)	0 (0)	1 (2)	0.683
Hyphema	1 (2)	0 (0)	1 (2)	0.683
Device touch to the iris	1 (2)	1 (5)	0 (0)	0.317
Device dislocation	1 (2)	1 (5)	0 (0)	0.317
One of the recorded complications	18 (30)	10 (53)	8 (20) *	0.015

* Significant difference between subgroups (*p* < 0.05). The chi–square test was used to compare subgroups. Clinically relevant hypotony was defined as intraocular pressure < 6 mmHg and was clinically relevant in case of choroidal effusion or requiring intervention; FB = Fornix based, LB = Limbus based, 25 or 27 = Gauge number for needle diameter.

## Data Availability

Data available upon reasonable request.
